# Hospital and Institutional News

**Published:** 1920-05-29

**Authors:** 


### HOSPITAL AND INSTITUTIONAL NEWS.

### LONDON UNIVERSITY.

Two facts concerning yet further activities of this university come to notice at the present time. The first is that the important steps which we detailed a few months ago, concerning the development of higher commercial education in London, are approaching completion, both by increasing the teaching facilities and by the institution of a new degree of Bachelor of Commerce (B. Com.). The second is the Government's offer of a site of 11½ acres behind the British Museum for the erection of central buildings as described in *The Times* of May 19-20 and 26. With regard to the former, the original appeal was made by the City Appeals Committee for £500,000, and at the present time more than half this amount has already been subscribed or promised, but in view of the great importance of the project and the continuing increase in prices it is not surprising that a second urgent appeal is now issued. Of its success we feel assured, but may here give a brief summary of the main channels into which funds received will now be diverted. The money is required to found new professorships and lectureships, to extend the building now available for training, to set up a bureau for students who cannot enrol at an institution but wish to study at home; to create up-to-date commercial literature; to give scholarships and to improve the libraries. In point of time the most urgent need is completion of the proposed extension of the existing buildings of the London School of Economics, Aldwych, where most of the commercial teaching will be given. Thus it is a point to note that the total sum required for the building being above £140,000, it so far has been possible to allot to this purpose only about £50,000 out of the money received. We have received a copy of the plans of the new buildings and also a list of subscribers of £100 and upwards. The former suggest excellent architectural designs, and the latter the imminence of further willing response to an appeal of the greatest national importance.

### A NEW WOMEN'S PAPER.

The proprietors of *Time and Tide* have the courage of their conviction that they have something good to offer the public when they choose the present moment of costly production for the appearance of the new weekly journal, *Time and Tide*. It is thought, to quote the words of Miss Lilian M. Faithfull, Principal of Cheltenham Ladies' College, that "there is an opening for a paper which will represent the many new interests that are coming into the lives of women, as well as those which have always been naturally associated with them," and a number of well-known people offer a cordial welcome in the first number to the new venture. A wide range of subjects is presented, including an article on the Bastardy Bill by Mrs. H. A. L. Fisher, one on that almost forgotten book of Fanny Burney's, "Eveline," by Alice Meynell, another on "Gardening for Women" by Viscountess Wolseley, a short comedy by Margaret Macnamara, and some wonderful verses by Gerald Gould. If the succeedings numbers come up to the first, fourpence will gladly be spent by many women, and certainly some men.

### ANOTHER RESEARCH APPEAL.

We would draw attention to the essentially sound decision on the part of the governing body of the Lister Institute, who have decided to make the requisite changes in their articles of association and are now proposing to establish a research hospital on a site adjacent to the institute. As is now more known through press publicity, the Council of Medical Research is the channel through which funds provided by the State, and devoted to the advancement of medicine by research, reach the Universities, research institutes, and individuals who are engaged in that work, and it is therefore primarily to this body that the appeal is addressed. Nevertheless we understand that the Director of the Lister Institute, Chelsea Gardens, would be glad to give any information desired to those who may feel inclined to take interest in forwarding the scheme.

### ORKNEY MEDICAL CENTENARIAN.

The doyen of the medical profession in the British Isles is Dr. James Scarth Spence Logie, who celebrated his centenary on the 11th instant. He is the son of a Scottish minister, who in the early half of last century had spiritual charge of several towns in the far north of this island. James Logie was born at Kirkwall on May 11, 1820; he received his early education at the Kirkwall Grammar School, commenced his medical course at the University of Edinburgh in 1837, became a licentiate of the Royal College of Surgeons in 1841, and took his M.D. at the early age of twenty-two in the year 1842. This progress was very rapid, but Logie had the advantage of studying under professors of world-wide reputation, and his diploma is signed by, amongst others, Sir Robert Christison, Professor of Materia Medica, and one of the most eminent of toxicologists; Professor James Syme, who was described as "the best and ablest surgeon Scotland ever produced"; Sir James Young Simpson, Professor of Midwifery, who discovered the anæsthetic virtues of chloroform; and by Dr. Thomas Stewart Traill, Professor of Medical Jurisprudence. During his long life Dr. Logie has witnessed many changes in his own profession and many changes in the Church of his fathers, in which his personal and family interest are keen. He has remained a general practitioner in urban districts all his life, and we are glad to know that in well-deserved rest and retirement his health, despite his advanced years, is of the best. Naturally, Dr. Logie has been the recipient on the occasion of his centenary of messages of congratulation from far and near.

### MOTORS FOR MEDICAL OFFICERS.

Many local authorities now provide medical officers with motors, realising that rapid transport enables them to get through much more work than otherwise could be the case. In some cases authorities make allowances where officers provide their own cars, and this applies in the County of Durham, where some of the district tuberculosis officers are now running their own cars. As Dr. Macrae, the central tuberculosis medical officer, reports to the County Council, on account of the capital expenditure entailed in the provision of cars, other medical officers are still without them, and are forced to waste time travelling by slow, stopping trains or waiting for train connections at isolated railway junctions. He mentions as an example that one medical officer, owing to inadequate railway facilities, could visit only two patients in a day, whereas by using his motor-car he is now able to see twelve people in the same time. The saving of time and the extra amount of work done are very important reasons for the use of motor-cars for tuberculosis work, and he suggests that the initial cost should be advanced on easy terms to medical officers to enable them to get cars.

### NEW CLINIC AT CARDIFF.

Last week a useful addition was made to King Edward VII. Hospital, at Cardiff, in the shape of a new psychiatric clinic. Experience has shown that patients in early stages of indisposition of mental and allied nature often yield to treatment in the early phases, and the clinic, which is in the charge of Dr. Edwin Goodall, is confidently expected to show good results. Patients will be seen in the same way as ordinary patients who might be attending for surgical, medical, or other kinds of treatment. This new development was announced at the monthly meeting of the board of management of the hospital, when it was resolved that the question of the location of a permanent nurses' home and the future development of the hospital and its financial prospects, which are giving some anxiety, should be referred to a special committee. One of the chief financial difficulties is the impossibility of making even an approximate estimate as to expenditure. As an instance, at King Edward VII. Hospital something like £14,000 to £15,000 has been spent on buildings in excess of last year's estimates.

### PRIVATE HOSPITALS AND HOMES IN SCOTLAND.

The *Glasgow Herald* has, in a recent issue, an excellent article on private hospitals and homes, and deals in a sensible way with the question of providing more accommodation in sickness for those whose means and social position are such as to be above the requirements of the ordinary voluntary hospital, but who can ill afford the fees necessarily required for board and residence in a private home which, being run on business principles, cannot provide much elasticity in the charges for accommodation. The writer of the article reviews what facilities of the kind there are at the moment, and shows that Scotland is as badly off as England in the matter of looking after what, for this purpose, may be described as the middle classes. In Edinburgh, Chalmers Hospital has about twenty beds allocated to paying patients with moderate incomes; eighteen of these are divided between two wards, partial privacy being effected by wooden screens placed, when required, around the beds. The managers of the Royal Infirmary have apparently under consideration the admission of paying patients, and recently a home has been opened for about fifty patients who can pay weekly charges varying from £2 to £4 10s. This home is in every way admirably equipped, and approaches as nearly as possible to meeting the varied surgical and medical requirements, only to be found efficiently supplied in our large public hospitals. But Glasgow is behind the times, and the only venture so far is represented by what is being done at the M'Alpine Home and the Women's Private Hospital in Lynedoch Place. If those who supply capital for such badly-needed accommodation are content with small profits, or if homes on a co-operative basis could be established for the use of members and their families, economically managed private hospitals and homes could without doubt be carried on without recourse to charity.

### GOLD IN HIS BOOTS.

The medical staff at Lambeth Infirmary have had one of those unusual experiences which help to enliven the often dull hours of infirmary medical service. A Pole travelling from America to Danzig was taken ill at a Y.M.C.A. hut near Waterloo Station, and was removed, apparently in an almost destitute condition to Lambeth Infirmary. An examination of his clothing, however, showed that 3s. in English money was not his sole wealth, for stitched away in the most unusual places were dollar notes to the value of 990 dollars, along with 22 gold ten-dollar pieces. His boots were exceptionally heavy, and from the corner of one of them a coin was protruding. The $x$-rays were requisitioned, with the result that between the leather of the soles of the two boots twelve more coins were discovered, presumably gold ten-dollar pieces. When he left the infirmary a few days' later his paper money and the twenty-two gold coins were returned to him, after the cost of his maintenance had been deducted, along with his two boots still containing the gold ten-dollar pieces.

### THE LATEST INEPTITUDE.

With that perversity which marks those who first cry for the moon and then complain because it is made of green cheese, the *Pall Mall Gazette* has been mentioning that the "amount" of the "State subsidy" for hospitals, which it seems less anxious now to advocate, is "mentioned" as two million a year. A suitable reference to the "already over-taxed and otherwise heavily burdened middle class" aptly followed, but it is suggested that its position might be eased, without reducing the burden, if the Exchequer compelled the hospitals to reserve "not less than one-third" of their beds for paying patients. This delusion Lord Knutsford did his best, we thought, to expose not many weeks ago; but State aid is like pitch: it is easier to handle than to get rid of. The nonsense talked of late in this connection by the lay Press, which largely assisted the

"scare" that it professes to chronicle, is beneath the consideration of those who know the facts.

### V.A.D.s AND DISTRICT NURSING.

Now that the county organisations are getting to work on behalf of the great scheme which accompanies the appeal of the Joint Council of the Red Cross and the Order of St. John, for the benefit of the voluntary hospitals, the future share which may fall to the V.A.D.s is receiving much attention. The Ministry of Health appears to look to them for such work as they may be fit to do, but the practical decision is being properly left to the County Medical Officers of Health. Dr. Middleton Martin, county M.O.H. for Gloucestershire, thinks a place can be found for such voluntary workers where the scheme is advanced far enough to see where they can help. He anticipates "a regular nursing service," and is fully aware that the district nursing in the county, as elsewhere, is understaffed. The decision will probably depend upon the organisation of the scattered though connected health centres. If these can be well organised the V.A.D. may find a useful place; for unskilled help is only dangerous when it is not subordinated to trained supervisors who may be glad to delegate simpler duties to subordinates only partly trained.

### WREXHAM APPOINTMENT.

Mr. Leslie W. T. Spencer, Assistant Secretary of the King Edward VII. Hospital, Cardiff, has been appointed Secretary at the Wrexham Hospital. Mr. Spencer has been for the last ten years on the office staff of the institution, and for the past five years as Assistant Secretary. He joined H.M.'s Forces during the War, served for three years, and was with the 15th Batt. Welsh Regiment in France. The numerous friends of Mr. Spencer all wish him well in his new sphere of activities.

### ST. DUNSTAN'S AND THE DERBY.

To see the Derby in comfort and incidentally to help the funds of St. Dunstan's Hostel for Blinded Soldiers and Sailors is the substance of a recently promoted scheme. A number of omnibuses will run from various theatres in London to the course at a charge of £3 per head. This sum will provide not only a comfortable journey to and fro, but a position in No. 1 upper carriage enclosure, a six-course lunch and tea in a reserved marquee.

### TEA-DRINKING INDIA.

It is obvious that if India became a tea-drinking country, as England is, she could consume the whole of her present production of the leaf. That consummation may be far distant, but there is evidence that the number of persons in India who drink tea is steadily increasing. In the annual report on tea production issued from the Department of Statistics, it is estimated that the total internal consumption last year was about 50,000,000 lb., which compares with 42,000,000 lb. in 1917-18, and an average of 36,000,000 lb. for the last five years. The method of encouraging the use of tea by selling it in prepared liquid form in shops is having a considerable effect, and the popularity of the beverage is shown by the opening of a large number of these shops during the past year or two.

### CHILDREN'S COURTS.

The measure shortly to be introduced into the House of Lords and having among its objects the centralisation of juvenile courts in London, is not being given a very friendly reception in advance, for reasons which appear difficult to answer with much effect. The Bill makes provision for the closing of existing courts and the setting up of a central court under the presidency of a police magistrate with two other justices, one of whom shall be a woman. The main objection to this centralisation is that it is foreign to the whole atmosphere and purpose of the juvenile courts which, when properly run, have done excellent work. It removes the more or less paternal exercise of correction, and gives place to the strict police court discipline which seems inevitable under a stipendiary magistrate. There would be a standardisation in the treatment of young offenders, which is precisely what should be avoided. There is also bound to be a greater show of ritual, together with more awesome circumstances that are not present in the local courts. The mere fact of taking children from all parts of the Metropolitan police district to the central court makes a greater occasion of it, and excludes the personal influences as exercised locally. Women probation officers strongly oppose the measure, and several magistrates have expressed disapproval. It is an important question, for it takes so little for a young delinquent to become a criminal; but the case of the promoters and supporters of the Bill remains to be heard, and until then it would perhaps be wise to suspend final judgment.

### CHILD WELFARE IN INDIA.

Almost suddenly the question of giving the children a fair chance has appealed to the Indian public. This may be attributed in part to the change rapidly taking place in the conditions of life in India. In the Indian village, given space, open air, and sunshine, the lack of knowledge was made up for by the lack of microbes, but since the tendency to live in towns grew overcrowding has greatly increased the risks by which the young Indian children are surrounded. Educated Indians are now fully aware of the serious state of things attending early childhood in India, as evidenced by the movements springing up on all sides to form societies to cope with it. The movement to promote child welfare is assuming such large dimensions that it is now the most prominent feature in the public health administration of every Indian town of any importance. European employees of labour are beginning to participate in the movement, and at the recent child welfare exhibition at Delhi an exhibit of interest was shown by Messrs. Cooper Allen and Company (Cawnpore), of welware work carried on among the employees of the North-West Tannery Company, Cawnpore. It constituted an excellent example of the way in which captains of industry in India recognise their responsibilities to their employees, and should give a lead to local bodies in the organisation of child welfare work.

### CONFIDENTIAL "V.D." NOTIFICATION.

Dr. Franks, the Venereal Diseases Medical Officer, reporting to the Durham County Council, says there is still difficulty in getting patients to attend the clinics for a sufficient time to complete the treatment, or arrest the disease, and he is convinced that this position is insurmountable until some form of confidential notification is adopted.

### THOSE STUDENTS.

The Health Committee of the Willesden Urban District Council reports that it has considered a letter from the health visitors protesting against being required to conduct the students from King's College on their daily rounds of business and to demonstrate their duties to them. The Committee carefully considered this letter, and expresses the opinion that so far as the Council is committed at present the arrangements should be carried out, adding that in the event of any further applications being received from King's College the Committee proposes to reconsider the position.

### THE OUT-PATIENTS AT MIDDLESEX HOSPITAL.

The Chairman of the Middlesex Hospital, the Earl of Athlone, is making a strong appeal for funds to remodel the out-patient department, which is no longer adequate to meet the requirements made upon it. During the last complete year the number of attendances have risen to over 166,000, and in addition there were 37,000 attendances in the electrical department. There are thus 200,000 attendances, as compared with the 128,000 a few years ago. The exact sum required is not stated, but it must be large. The appeal has been started by that rather agitating proposition, the conditional donation. Mr. William Hartmann, who is a generous supporter of many hospitals, has offered £5,000 to the fund if five other donors will each give the same amount before June 1. This is a sporting offer, and we hope that the friends of Middlesex Hospital will take it up.

### A PLAN OF CAMPAIGN AT CHICHESTER.

The governors of the Royal West Sussex Hospital are to be congratulated upon having a balance in hand at the end of their financial year. It is a small balance of £35—but it is a balance none the less. The hospital at Chichester received wounded soldiers, and during the year a grant of £2,000 came from the British Red Cross Society, and £500 from the Midhurst Farmers' Red Cross Sale Committee. These and the other sources of income, coupled with strict economy, helped to bring about this happy state of affairs. But when these grants cease to be received a big effort will be necessary to keep the institution free from debt, with outgoings at £9,000 per annum instead of £5,000 as in pre-war days. Publicity is gladly given to the special appeal that is being made to the residents in the district to increase the ordinary income of the hospital, preferably in annual subscriptions. A call for payment by patients is in the air, for it is realised that something will have to be done to make both ends meet. Here, again, the need is rather to develop existing sources of income to the full than to create new ones, for the plan of sectional subscriptions from every class served by a hospital is in germ everywhere. Only enthusiasm and brains are needed to develop it methodically.

### ANOTHER HOSPITAL MOVING.

According to a report of the Hertfordshire Medical Officer of Health, the Alexandra Hospital for Crippled Children at Queen Square, which was established in 1867, will shortly be moved to new premises on the hills at Caversham, near Reading. The hospital will provide accommodation for children, young persons, and adults suffering from tuberculous disease of the bones and joints. In the first instance the hospital will be used for fifty discharged sailors and soldiers; twenty-five children under five; seventy-five children from five to sixteen; twenty-five young persons from sixteen to twenty-one; and twenty-five private contributing patients under sixteen, making a total in all of 200 beds. Not only treatment, but education and instruction will be given, and (we understand) the estimated cost of maintenance will be two guineas a week per patient.

### ASYLUM MEDICAL OFFICERS.

York City Councils Health Committee reports receipt of a communication from the Board of Control with suggestions for improving the standard of training of assistant medical officers of asylums and making the service more attractive, as by the grant of study leave from time to time. The Committee has informed the Board that whilst it is not prepared to incur expense in the matter, it would afford all reasonable facilities for the assistant medical officer attending (at his own expense) any training centre for improving his position in the profession.

### THIS WEEK'S DRUG MARKET.

Business in the drug market is more or less at a standstill, partly in consequence of an unofficial extension of the Whitsuntide holiday, and partly because this market is involved in the general pause in trade which has come so suddenly and was not foreseen by the vast majority of people. For many months business in drugs and chemicals had been exceptionally active, on account of orders coming in from all parts of the world. Instead of a large volume of orders, dealers are in many cases receiving cables cancelling the orders, and in the circumstances it is not surprising that a feeling of depression is strongly in evidence. Our opinion is that this condition will not last long, and that we may expect a revival both of home and overseas trade in the early future, but, in all probability, at a lower level of prices and on a more stable basis. In the temporary absence of business on anything like a large scale prices are more or less nominal. Our own view is that hospital buyers would be well advised to adopt the tactics of large-scale operators and play the waiting game.

